# Early diagnosis of psoriatic arthritis among psoriasis patients: clinical experience sharing

**DOI:** 10.1007/s10067-020-05132-1

**Published:** 2020-05-28

**Authors:** Yu-Jih Su

**Affiliations:** Rheumatology, Allergy and Immunology Division, Kaohsiung Chang Gung Memorial Hospital, Chang Gung University College of Medicine, 123 Ta Pei Road, Kaohsiung, Taiwan

**Keywords:** Allergic rhinitis, Metabolic syndrome, MicroRNA, Psoriasis, Psoriatic arthritis

## Abstract

**Background:**

The early detection of psoriatic arthritis (PSA) poses a challenge to rheumatologists, even when their diagnosis is aided by sonography. In order to facilitate early detection of PSA among patients with psoriasis (PSO), we retrospectively analyzed of the relationships between serological markers and comorbidities in 629 psoriatic patients, 102 of which had PSA, while the other 527 had PSO.

**Results:**

Serological markers were found not to be useful in distinguishing between PSA and PSO (*p* > 0.05 for all comparisons). The prevalence rate of PSA among PSO patients was around 19.4%. Two components of metabolic syndrome—hyperlipidemia (2.94%) and gout (4.9%)—were significantly more prevalent in PSA patients than in PSO patients (*p* < 0.05). The odds ratio for PSA is 15.94 in patients with hyperlipidemia with a 95% confidence interval (CI) of 1.64–154.80; meanwhile, the odds ratio for PSA is 3.83 in patients with gout with a 95% CI of 1.19–12.31. Allergic rhinitis (5.88%) was more prevalent in PSA patients than in PSO patients (*p* < 0.01). The odds ratio was 8.17 in patients with allergic rhinitis with a 95% CI of 2.26–29.50. Plasma hs-miR-210-3p distinguishes PSA from PSO, and its levels can also be distinguished from PSA after treated with anti-TNFα biologics agents (both *p* < 0.05).

**Conclusions:**

No clinical available serology markers, but hyperlipidemia, gout, axial spondylopathy (inflammatory back pain), or allergic rhinitis, could differentiate between psoriatic arthritis from psoriasis. Plasma hs-miR-210-3p and comorbidities may differentiate psoriatic arthritis from psoriasis.

**Table:** 

**Key Points** *• Clinical manifestations and comorbidities are different between psoriatic arthritis and psoriasis only patients.* *• Traditional serology markers are similar between psoriatic arthritis and psoriasis-only patients.* *• Plasma hs-miR-210-3p distinguishes PSA from PSO, and its levels can also be distinguished from PSA after treated with anti-TNFα biologics agents in our study.*

**Electronic supplementary material:**

The online version of this article (10.1007/s10067-020-05132-1) contains supplementary material, which is available to authorized users.

## Background

The early detection and diagnosis of psoriatic arthritis (PSA) pose a challenge to rheumatologists [1], even when aided by sonography [2]. The MAdrid Sonographic Enthesitis Index (MASEI) has a sensitivity of only 30% for diagnosing PSA. An update of the treat to target concept of a Canadian dermatologic expert suggested looking beyond the skin [3]. Such treat to target concept requires the early diagnosis and treatment of PSA [4, 5]. Using the International Classification of Diseases, Ninth Revision, Clinical Modification (ICD-9-CM) diagnostic codes in a hospital database, we retrospectively identified and analyzed the medical records of all patients with psoriasis (PSO) or PSA but without any autoimmune diseases. To support our proposed concept, we carried out a preliminary study and prospectively compared more than 150 plasma microRNAs from four PSA patients with four lupus patients, three osteoarthritis (OA), and three PSO patients. One recent study reported that the prevalence rate of PSA is about 17% among PSO patients in dermatology clinics [6]. By comparing the relationships between serological markers and comorbidities with PSO and PSA, we attempted to identify markers and clinical characteristics that may aid the early diagnosis of PSA among PSO patients. We further demonstrated in our preliminary study that the clinical presentation may hint at serology and pathophysiology diagnosis.

PSO is a systemic disease that involves the skin [7], joints [8, 9], nails, kidneys [10], vascular system [11], and heart [12] and may be related to the development of cancer [13]. A previous large retrospective study using the National Insurance database indicated that PSO has dozens of comorbidities when compared to normal controls [14]. Other previous studies have shown that PSO and PSA are difficult to diagnose based on serological findings; however, only one report focuses on using autoantibodies against extractable nuclear antigens (anti-ENA) in PSO [15], but none has focused on it in PSA patients. We collected all available data on clinical anti-ENA antibody status among PSO and PSA patients and investigated the correlations between disease status and anti-ENA results. Furthermore, we gathered information on the spectrum of clinical conditions that may be associated with PSO, including dermatitis [16], pyoderma [17, 18], diabetes [19, 20], hypertension [21], hepatitis [22, 23], gout [24, 25], axial spondylopathy [26], dyslipidemia, allergic rhinitis, bronchitis, hepatitis B virus (HBV) carrier status [27], and previous hepatitis C infection among anti-HCV–positive persons [28].

In this prospective proof-of-concept preliminary study, plasma was collected from patients with osteoarthritis (OA), PSO, PSA, and SLE for microRNA analysis. Serology microRNA arrays can reflect some inflammatory pathways in several aspects, instead of just the very limited clinical tests currently available. Since the current situation has limited clinical tests available, the aim of this study is to find a reference for selecting particular PSA patients for earlier clinical diagnosis.

## Methods

### Patients

The first part of our study is a retrospective chart review that describes data from all patients over the age of 20 years who had been diagnosed with PSO or PSA between July 1, 2000 and December 31, 2014 at Chang Gung Memorial Hospital, Kaohsiung (CGMH-KS) Medical Center. CGMH-KS is a tertiary care referral center located in Kaohsiung County, which is located in southern Taiwan, and serves a population of approximately two million people. We excluded any patients with a concurrent diagnosis of another autoimmune disease, including systemic lupus erythematosus, systemic sclerosis, sicca syndrome, dermatomyositis, and polymyositis. The study was carried out using a protocol approved by the Ethics Committee of Chang Gung Memorial Hospital (Institutional Review Board numbers: 102-4669B and 104-5733B). Informed consent was not obtained from the individual patients in the retrospective part of study. Prior to analysis, the medical records/data were anonymized and de-identified.

The second prospective study only has male patients which were divided into several subgroups: four male patients with PSA, three male patients with PSO, four male patients with SLE (as disease controls), and three male patients with on osteoarthritis (OA, as non-inflammatory controls). We collected plasma from all these patients for microRNA array analysis. We obtained informed consent from each patient, and the study was conducted according to a protocol approved by the Ethics Committee of Chang Gung Memorial Hospital (Institutional Review Board number: 104-5733B).

### Data collection

Patients’ demographic and clinical characteristics were analyzed, including age, gender, leukocyte differential count, hemoglobin, hematocrit, platelet count, creatinine, uric acid, C-reactive protein, erythrocyte sediment rate, rheumatoid factor, lipid profile, anti-nuclear autoantibodies, anti-ENA autoantibodies, anti-phospholipid autoantibodies, anti-neutrophil cytoplasmic autoantibodies, anti-citrullinated protein antibodies (ACPA), hepatitis B surface antigen, anti-hepatitis C antibody, and comorbidities (diabetes, hypertension, gout, axial spondylopathy, asthma, bronchitis, allergic dermatitis, etc.).

### MicroRNA preparation

Samples were centrifuged at 1000×*g* for 10 min to pellet cellular debris, and we used the supernatant for RNA extraction. We extracted total RNA from 300 μL fluids using the miRNeasy kit (Qiagen) as previously described by Weber et al. [29]. All the microRNA levels were confirmed with real-time PCR with PanelChip™ qPCR on QuarkBio PanelStation™.

### Statistical analysis

Patient characteristics were reported as simple descriptive statistics. In univariate analysis, categorical variables were compared using Fisher’s exact test, while continuous variables were compared using the Mann–Whitney *U* test for non-parametric data with a non-normal distribution and the *t* test for normally distributed data. MicroRNA plasma levels were calculated between groups using ANOVA and post hoc analysis with the Bonferroni method. We performed multivariate analysis by estimating odds ratios (ORs) and 95% confidence intervals (CIs) using a logistic regression model. The chi-square test was used to compare different diseases between each group and examine the prevalence of comorbidities between PSO and PSA patients according to level of disease severity. Statistical significance was defined as a *p* value of less than 0.05. All analyses were performed using the SPSS software program, version 15.5 (SPSS, Chicago, IL).

## Results

### Demographic characteristics of patients

In total, 629 patients (102 with PSA and 527 with PSO) were identified during the period of July 1, 2000 through December 31, 2014. PSA diagnoses were confirmed by chart review or the presence of a relevant ICD-9-CM code diagnosed by a rheumatologist. The prevalence rate of PSA among PSO patients was 19.35%.

The two subgroups did not significantly differ with regard to total leukocyte count, platelets, hemoglobin, hematocrit, lipoprotein profile, inflammation markers (C-reactive protein, erythrocyte sediment rate), titers of anti-nuclear antibodies, or rheumatoid factor (*p* > 0.05 for all comparisons) (Table 1).

**Table 1:** Demographic data of patients in this study (*N* = 629)

	All patients (*N* = 629)	Psoriatic arthritis (*n* = 102)	Psoriasis (*n* = 527)	*p* value^ζ^
Age (years)	46.64 ± 14.82	44.83 ± 15.08	47.00 ± 14.76	0.19
Leukocytes (1000/μL)	8.22 ± 4.14	7.80 ± 2.63	8.30 ± 4.37	0.30
Neutrophils (%)	65.92 ± 11.79	64.58 ± 13.06	66.15 ± 11.56	0.32
Lymphocytes (%)	25.36 ± 10.63	26.36 ± 11.70	25.19 ± 10.44	0.41
Monocytes (%)	6.03 ± 2.08	6.28 ± 1.68	5.99 ± 2.14	0.3
Platelets (1000/μL)	251.73 ± 97.93	265.82 ± 101.43	248.91 ± 97.09	0.14
Hemoglobin (g/dL)	13.50 ± 2.47	13.40 ± 1.70	13.52 ± 2.61	0.71
Hematocrit (%)	40.23 ± 5.21	40.31 ± 4.53	40.21 ± 5.35	0.90
High-density lipoprotein cholesterol (mg/dL)	50.82 ± 16.12	50.70 ± 12.78	50.85 ± 16.91	0.96
Low-density lipoprotein cholesterol (mg/dL)	104.35 ± 32.32	108.25 ± 30.08	103.37 ± 32.89	0.42
Triglyceride (mg/dL)	133.71 ± 85.16	132.88 ± 72.19	133.89 ± 87.88	0.95
Rheumatoid factor (IU/mL)	11.56 ± 7.16	10.95 ± 4.23	11.84 ± 8.16	0.44
Creatinine (mg/dL)	0.89 (0.71, 1.08)	0.89 (0.76, 1.1)	0.89 (0.7, 1.07)	0.51
Uric acid (mg/dL)	6.1 (5.0, 7.4)	5.75 (4.6, 7.6)	6.2 (5.1, 7.3)	0.54
C-reactive protein (mg/L)	6.2 (1.59, 22.05)	5.5 (2.02, 24.02)	6.3 (1.47, 21.35)	0.86
Erythrocyte sedimentation rate (mm/h)	16 (7, 33)	16 (7, 33)	16 (7, 31.25)	0.49
Anti-nuclear antibody (dilution)	40× (40×, 40×)	40× (0, 40×)	40× (40×, 40×)	0.10

^ζ^Compare between the psoriatic arthritis subgroup and the psoriasis subgroup. Anti-nuclear antibody (dilution), 40× means 1:40 dilution

Mann-Whitney test for creatinine, uric acid, C-reactive protein, erythrocyte sedimentation rate, and anti-nuclear antibody, which are expressed as median (interquartile range); the rest was calculated with *t* test, which are expressed as mean ± standard deviation

### Anti-ENA autoantibodies, anti-phospholipid autoantibodies, and anti-neutrophil cytoplasmic autoantibodies in PSO and PSA patients

Data regarding anti-ENA levels were available for 41 patients (18 with PSA and 23 with PSO). We reviewed charts to determine levels of anti-ENA autoantibodies (including anti-Ro, anti-La, anti-U1 RNP, anti-Sm, anti-Scl 70, and anti-Jo 1), anti-phospholipid autoantibodies (anti-beta 2 glycoprotein 1 and anti-cardiolipin IgG or IgM), anti-neutrophil cytoplasmic autoantibodies, and ACPA. Anti-Sm and anti-U1 RNP were significantly higher in PSO patients (*p* < 0.05). However, none of the aforementioned autoantibodies was independently associated with PSO after multivariate logistic regression analysis (*p* = 1.00) (Supplementary Table 1).

### Comparison of the correlation coefficiencies between psoriasis comorbidities and psoriatic arthritis comorbidities

Using ICD-9-CM diagnostic codes, we analyzed each of the relationships between hypertension, gout, axial spondylopathy, asthma, bronchitis, allergic dermatitis, and pyoderma with PSO or PSA status (Table 2). Various diseases were happening significantly higher in PSA than in PSO, including hyperlipidemia, gout, axial spondylopathy, and allergic rhinitis (*p* < 0.05) (Table 2). The odds ratios for a patient with PSA to get any comorbidity of the following diseases, comparing to a patient with PSO, were listed as the following: 15.94 times to have hyperlipidemia with a 95% confidence interval (CI) of 1.64–154.80, 3.83 times to have gout with a 95% CI of 1.19–12.31, 1.12 times to have axial spondylopathy with a 95% CI of 1.05–1.20, and 8.17 times to have allergic rhinitis with a 95% CI of 2.26–29.50 (Table 2).

**Table 2:** Correlations of other diseases with psoriasis and psoriatic arthritis disease status

*N* = 629	Psoriatic arthritis (*n* = 102)		Psoriasis (*n* = 527)				
	*n*	%	*n*	%	*p*-value^ζ^	Odds Ratio	95% C.I.
Dermatitis	12	11.76%	57	10.82%	0.73	1.10	0.57–2.13
Pyoderma	1	0.98%	16	3.04%	0.33	0.32	0.04–2.41
Hypertension	4	3.92%	7	1.33%	0.09	3.03	0.87–10.55
Hyperlipidemia	3	2.94%	1	0.19%	0.02*	15.94	1.64-154.80
Gout	5	4.90%	7	1.33%	0.03*	3.83	1.19-12.31
Axial spondylopathy	11	10.78%	0	0.00%	<0.01*	1.12	1.05-1.20
Allergic rhinitis	6	5.88%	4	0.76%	<0.01*	8.17	2.26-29.50
Chronic bronchitis	1	0.98%	1	0.19%	0.30	5.21	0.32–83.94
Hepatitis (non-B, non-C)	3	2.94%	5	0.95%	0.13	0.93	0.40–2.16
Hepatitis B carrier	4	3.92%	21	3.98%	1.00	1.05	0.33–3.30
Hepatitis C infection	5	4.90%	20	3.80%	0.79	1.16	0.41–3.28

*indicates *p* < 0.05 by Chi square

### Heatmap of PanelChip™ qPCR on QuarkBio PanelStation™ and comparison between PSA and SLE patients in the prospective proof-of-concept preliminary study

**Fig. 1:**
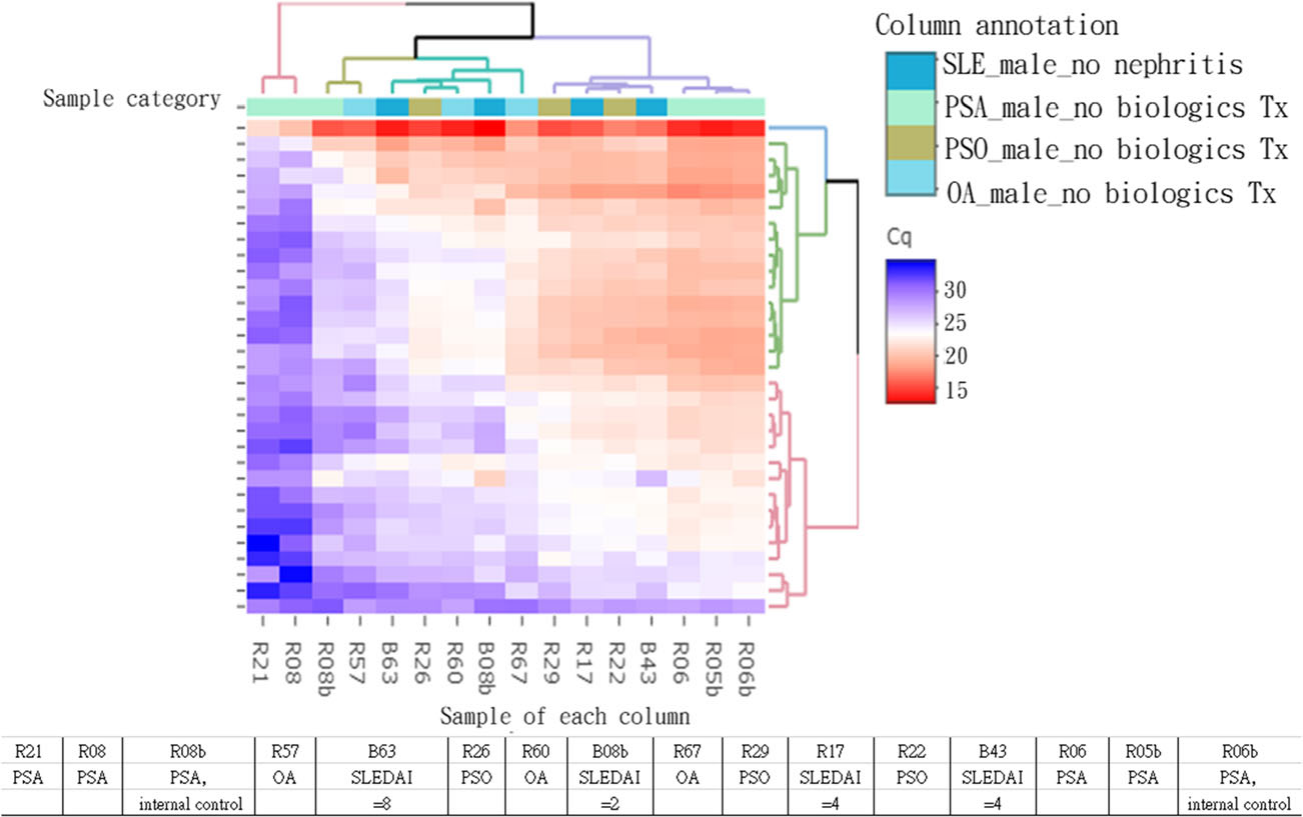
Result of heatmap of PanelChip™ qPCR on the QuarkBio PanelStation™

The resulting heatmap of PanelChip™ qPCR on the QuarkBio PanelStation™ is shown in Fig. 1. The heatmap includes the distribution patterns of the three different diseases, as well as OA as the non-inflammatory controls. The diseases include PSO, PSA, and SLE, and the heatmap clustering shows all samples based on microRNA expression pattern.

**Fig. 2:**
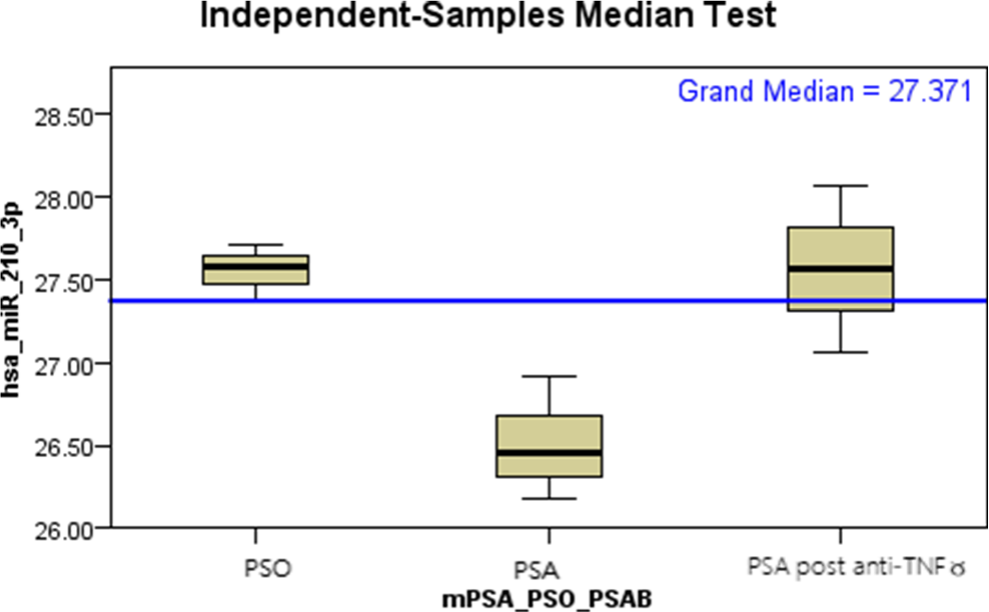
Treatment with anti-TNFα agents significantly increased hs-miR210-3p Ct levels in male PSA patients (*p* = 0.04)

We compared 128 plasma microRNA Ct levels between male PSO, male PSA, and male PSA patients. The Ct of hs-miR210-3p is statistically lower in male PSA patients, before biologics treatment, compared to male PSO patients (*p* = 0.04) (Fig. 2; Table 3). The Ct levels of hs-miR210-3p in male PSA patients were comparable with male PSO patients after they were treated with anti-TNFα agents. Treatment with anti-TNFα agents significantly increased hs-miR210-3p Ct levels in male PSA patients (*p* = 0.04) (Fig. 2; Table 3).

**Table 3:** Compare hs-miR-210-3p between psoriasis (PSO), psoriatic arthritis (PSA), and PSA post-anti-TNFα biologics agent treatment

**ANOVA**	**Multiple comparisons**	**Bonferroni**			
**hsa-miR-210-3p**	**Sum of squares**	**df**	**Mean square**	**Sig.**	
Between groups	2.181	2	1.090	.022*	
Within groups	.844	6	.141		
Total	3.024	8			
**Compared between 3 groups (*N*** **= 9)**		**Std. error**	**Sig.**	**95% Confidence interval**	
				**Lower bound**	**Upper bound**
PSO (*n* = 3)	PSA-NO biologics	0.306	0.044*	0.034	2.047
	PSA-POST biologics	0.306	1.000	− 1.014	0.998
PSA-NO biologics (*n* = 3)	PSO	0.306	0.044*	− 2.047	− 0.034
	PSA-POST biologics	0.306	0.042*	− 2.055	− 0.042
PSA-POST biologics (*n* = 3)	PSO	0.306	1.000	− 0.998	1.014
	PSA-NO biologics	0.306	0.042*	0.042	2.055

Biologics indicate anti-TNF-alpha therapy

*CI* confidence interval, *Std.* standard, *PSA* psoriatic arthritis, *PSO* psoriasis, *hsa Homo sapiens*, *miR* microRNA

*p < 0.05

## Discussion

The under-diagnosis of PSA among persons with psoriasis not only delays treatment but also increases the risk of joint damage [30]. In one study, the rate of PSA under-diagnosis was estimated to be about 9% [31]. Early diagnosis of PSA could also increase awareness in doctors to treat comorbidities. Upon comparing PSO and PSA patients, a Turkish study found that hypertension was significantly more prevalent in PSA patients [32]. Our present results also show a higher prevalence rate of hypertension in PSA patients (3.92%) than in PSO patients (1.33%), but the difference did not reach statistical significance (*p* = 0.09 with odds ratio 3.03, 95% CI 0.87–10.55). The hazard ratio of hypertension was higher for PSA than for PSO in one large US cohort study [24]. When talking about gout, our results are in line with previous study [24], with an odds ratio of 3.83 and a 95%CI of 1.19–12.31 for PSA, which is significantly higher than PSO patients (*p* = 0.03).

Coronary heart disease and metabolic syndrome are major comorbidities of PSA [33]. A large retrospective study in Europe reported that PSA was associated with dyslipidemia, diabetes, hypertension, axial spondylopathy, and rheumatoid arthritis [34]. We obtained similar results, which have an odds ratio of 3.83 and a 95% CI of 1.19–12.31 for PSA compared to PSO in patients with gout (*p* = 0.03), an odds ratio of 15.94 and a 95% CI of 1.64–154.80 for PSA compared to PSO in patients with hyperlipidemia (*p* = 0.02), and an odds ratio of 1.12 and a 95% CI of 1.05–1.20 for PSA compared to PSO in patients with axial spondylopathy (*p* < 0.01).

Psoriasis has been associated with non-alcoholic fatty liver disease in a prior meta-analysis [35]. In the present study, the prevalence rate of non-B, non-C hepatitis was higher among PSA patients (2.94%) than PSO patients (0.95%), but the difference did not reach statistical significance (*p* = 0.13) (Table 1).

The prevalence rate of allergic rhinitis was significantly higher in patients with PSA than in those with PSO (*p* < 0.01). The odds ratio is 8.17 with a 95% CI of 2.26–29.50 for PSA compared to PSO in patients with allergic rhinitis (*p* < 0.01). A previous large cohort study from Taiwan compared 51,800 PSO patients with normal controls using the National Insurance database and similarly demonstrated that PSO has a significant association with allergic rhinitis [14]. PSO and PSA are likely both associated with allergic rhinitis according to the aforementioned PSO study [14] and our current report (Table 2).

Allergic diseases are Th2-polarized diseases [36], whereas psoriasis is a Th17 disease, in general speaking, but could be a Th9-polarized disease in the gut [36, 37]. This transition may explain why allergic skin disease does not coexist with PSO in our cohort, but whether the Th9 links between the Th2 [38] and Th17 [39] pathogeneses need to be further investigated. Another explanation is that IL17E, also known as IL25, could propagate production of IL-4 and IL-13 in different organs, which stimulate the expansion of eosinophils [40], and link between allergic disease and psoriatic disease. Psoriatic arthritis has only few biomarkers [41, 42], but we failed to identify in anti-ENA markers (Supplementary Table 1).

In this prospective proof-of-concept preliminary study, we collected plasma from three male PSO, four male PSA, three male OA, and four male SLE patients for microRNA analysis. The distribution pattern of PSA in the microRNA array is divided into right and left parts in the heatmap (Fig. 1). Among the right part, these PSA patients mostly have simple clinical symptoms, such as arthritis, without other systemic diseases, and their clinical situations are very similar to lupus patients B043 and R017 with relatively low disease activity (SLEDAI = 4). The left-part PSA patients have more complex systemic diseases. The distribution pattern of the microRNA from the right-part PSA patients in the heatmap is closer to a normal control patient with moderate allergic rhinitis (R057). The miR210-3p was already known to be one of the markers in cell cycle regulation, cell survival, differentiation, angiogenesis, as well as in metabolism [43]. The miR-210-3p is upregulated in most solid tumors, and its levels correlate with a negative clinical outcome [44]. The miR-210 is a marker associated with bone [45]. More interestingly, it is a marker associated with nerve [46]. Since the difference and early acknowledge of arthritis between the PSA and the PSO patients could be patient awareness of pain, we decided to look into the miR210-3p in this preliminary research.

By combining the retrospective chart review and the prospective research results, we demonstrated in this research that the PSA patients could have either simple or complex clinical manifestations, including allergic rhinitis, gout, hypertension, dyslipidemia, and metabolic syndrome.

The search for useful clinical markers for distinguishing between PSO and PSA has been tedious. So, we ultimately turned into the microRNA chip to screen for potential serology markers to distinguish PSA from PSO. As a result, plasma hs-miR210-3p was found to be a marker that distinguishes patients with PSO from patients with PSA, as it changes significantly in Ct levels (*p* = 0.04). After treatment with biologics agents, the Ct level of hs-miR210-3p gradually increased and reached the average Ct levels of hs-miR210-3p as in PSO patients. The change between PSA patients and PSA patients after biologic treatment differed greatly (*p* = 0.04), which indicates that PSA patients’ hs-miR210-3p was restored after treatment (Fig. 2; Table 3).

This preliminary proof-of-concept research combined a retrospective chart review study and a prospective microRNA study. Still, the study has several limitations. First, some data were missing, and no longitudinal data from follow-up examinations were available. Second, steroid doses may have fluctuated in this cross-sectional study; in fact, PSO patients seldom receive steroid treatment. Third, several lifestyle variables and diseases associated with arthritis were not recorded, such as smoking [47], inflammatory bowel disease [48], and alcohol consumption [49–52], which might be associated with serology markers. Nevertheless, we still have some positive finding that help physicians to make early diagnosis of PSA among psoriasis patients by looking for comorbidities with gout, hyperlipidemia, inflammatory back pain, and allergic rhinitis. Moreover, miR-210-3p could be a marker that differentiates between PSO and PSA and also between before and after biologics treatment.

## Conclusions

Clinicians should be particularly aware of such manifestations as gout, hyperlipidemia, axial spondylopathy (inflammatory back pain), and allergic rhinitis in PSO in order to ensure the early diagnosis of PSA among psoriasis patients. Moreover, miR-210-3p could be a marker that differentiates between PSO and PSA and also between before and after biologics treatment.

## Electronic supplementary material

ESM 1 (DOCX 15 kb)

## Data Availability

All of the underlying research material related to our article can be accessed on demand by e-mail notification.
