# Supplementary material for: Early diagnosis of psoriatic arthritis among psoriasis patients: clinical experience sharing
Source: Clin Rheumatol. 2020 May 28;39(12):3677–84. doi: 10.1007/s10067-020-05132-1 (PMC7648743; doi:10.1007/s10067-020-05132-1)
Supplement: Supplementary file 1 — (DOCX 15 kb) [file 10067_2020_5132_MOESM1_ESM.docx]

Supplementary Table 1. Correlations of anti–extractable nuclear antibodies with psoriasis and psoriatic arthritis disease status.

|  | All patients (n=41) | Psoriatic arthritis (n=18), | Psoriasis (n=23), | univariate  p-value^ζ^ | multivariate  p-value^ζ^ |
| --- | --- | --- | --- | --- | --- |
| B2GP1 | 0 ( 0.00, 0.00 ) | 0 ( 0.00, 0.00 ) | 0 ( 0.00, 1.10 ) | 0.07 |  |
| Cardio IgG | 0 ( 0.00, 0.00 ) | 0 ( 0.00, 0.00 ) | 0 ( 0.00, 1.00 ) | 0.24 |  |
| Cardio IgM | 0 ( 0.00, 0.00 ) | 0 ( 0.00, 0.00 ) | 0 ( 0.00, 0.00 ) | 0.41 |  |
| Anti-Ro | 0 ( 0.00, 0.10 ) | 0 ( 0.00, 0.03 ) | 0 ( 0.00, 0.20 ) | 0.39 |  |
| Anti-La | 0 ( 0.00, 0.10 ) | 0 ( 0.00, 0.03 ) | 0 ( 0.00, 0.10 ) | 0.38 |  |
| Anti-RNP | 0 ( 0.00, 0.00 ) | 0 ( 0.00, 0.00 ) | 0 ( 0.00, 0.80 ) | 0.02* | 1.00 |
| Anti-Sm | 0 ( 0.00, 0.00 ) | 0 ( 0.00, 0.00 ) | 0 ( 0.00, 0.10 ) | 0.02* | 1.00 |
| Anti-Scl70 | 0 ( 0.00, 0.00 ) | 0 ( 0.00, 0.00 ) | 0 ( 0.00, 0.00 ) | 0.07 |  |
| Anti-Jo1 | 0 ( 0.00, 0.00 ) | 0 ( 0.00, 0.00 ) | 0 ( 0.00, 0.00 ) | 0.71 |  |
| Anti-centro | 0 ( 0.00, 0.00 ) | 0 ( 0.00, 0.00 ) | 0 ( 0.00, 0.00 ) | 0.38 |  |
| ACPA | 0 ( 0.00, 0.00 ) | 0 ( 0.00, 0.00 ) | 0 ( 0.00, 0.00 ) | 0.89 |  |
| PANCA | 0 ( 0.00, 0.00 ) | 0 ( 0.00, 0.00 ) | 0 ( 0.00, 0.00 ) | 0.22 |  |
| CANCA | 0 ( 0.00, 0.00 ) | 0 ( 0.00, 0.00 ) | 0 ( 0.00, 0.00 ) | 0.22 |  |

IQR, interquartile range; B2GP1, anti–beta 2 glycoprotein 1; Cardio IgG, anti–cardiolipin IgG; Cardio IgM, anti–cardiolipin IgM; anti-RNP, anti-ribonucleoprotein U1; anti-centro, anti-centromere; ACPA, anti–citrullinated protein antibody; PANCA, perinuclear antineutrophil cytoplasmic antibody; CANCA, cytoplasmic antineutrophil cytoplasmic autoantibody; all the concentrations are measured in IU/mL.

* values indicate p<0.05

ζ Compared between the psoriatic arthritis subgroup and the psoriasis subgroup.
